# Supplementary material for: The gut microbiota promotes hepatic fatty acid desaturation and elongation in mice
Source: Nat Commun. 2018 Sep 14;9:3760. doi: 10.1038/s41467-018-05767-4 (PMC6138742; doi:10.1038/s41467-018-05767-4)
Supplement: Supplementary file 1 — Supplementary Information [file 41467_2018_5767_MOESM1_ESM.pdf]

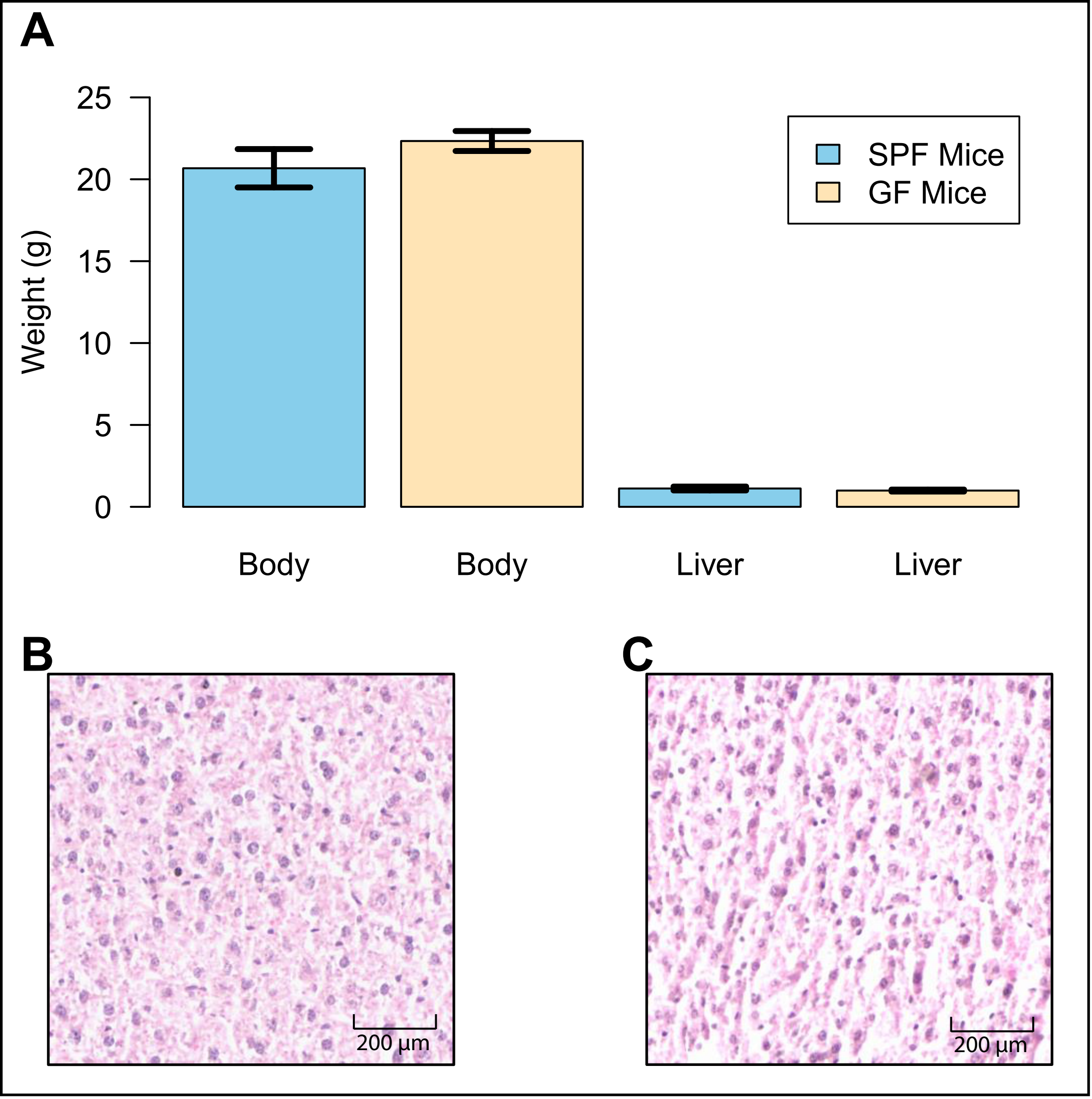

Supplementary Figure 1: Comparison of body/liver weight and liver histology.(A) Body and liver weight of SPF and GF mice (n=6/group). Representative images of hematoxylin and eosin (HE)-stained liver sections from SPF (B) and GF (C) mice. 20 × magnification, 10 × optical zoom. Error bars in the bar plots show the standard deviation.

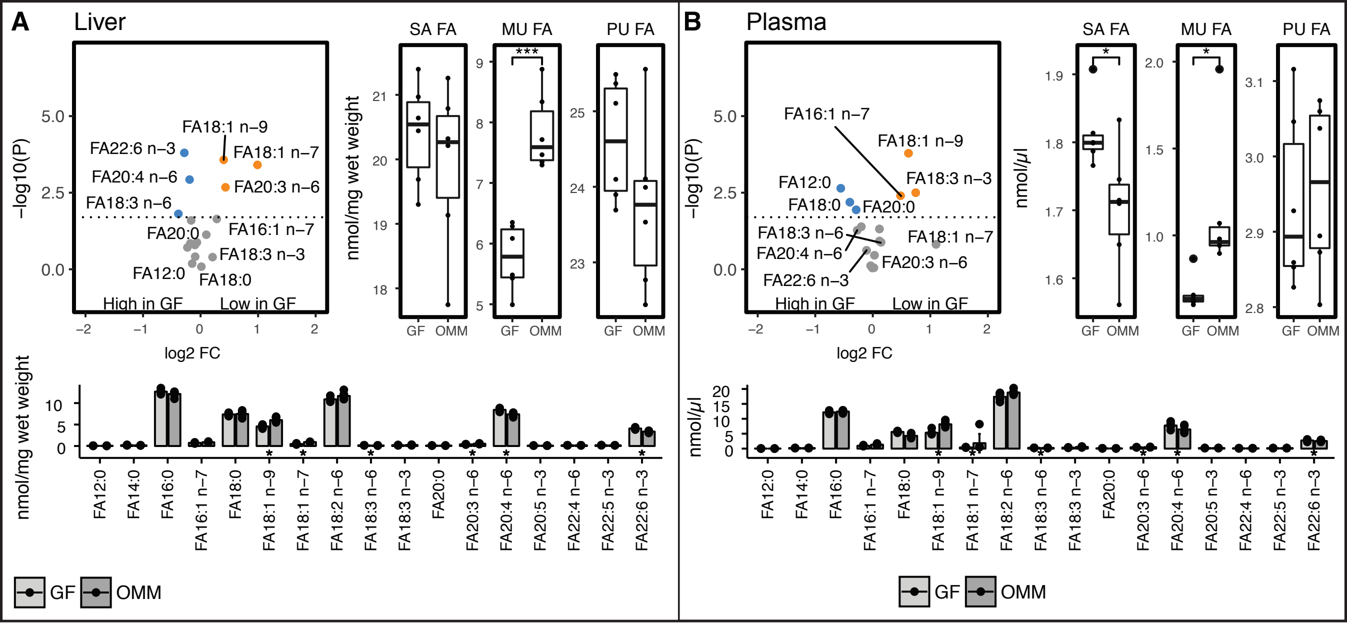

Supplementary Figure 2: Quantitative FA analyses of liver and plasma samples from GF and Oligo-MM^{12} **mice.** GF and Oligo-MM^{12} mice were fed a chow diet (n=6/group). (A) Total FA in liver and (B) plasma. Significance and log2 fold change in individual FA species shown in volcano plot; boxplots show concentrations of saturated (SA), monounsaturated (MU), and polyunsaturated (PU) FA species; individual FA levels of the different groups as barplots. In boxplots the thick lines represent the medians, the upper and lower lines of the boxes show the 25% and 75% quartiles and the whiskers are 1.5 times the interquartile range of the data. Error bars in the barplots show the standard deviation. FDR, false discovery rate. GF, germfree; OMM, Oligo-MM12. *p<0.05, **p<0.01, ***p<0.001.

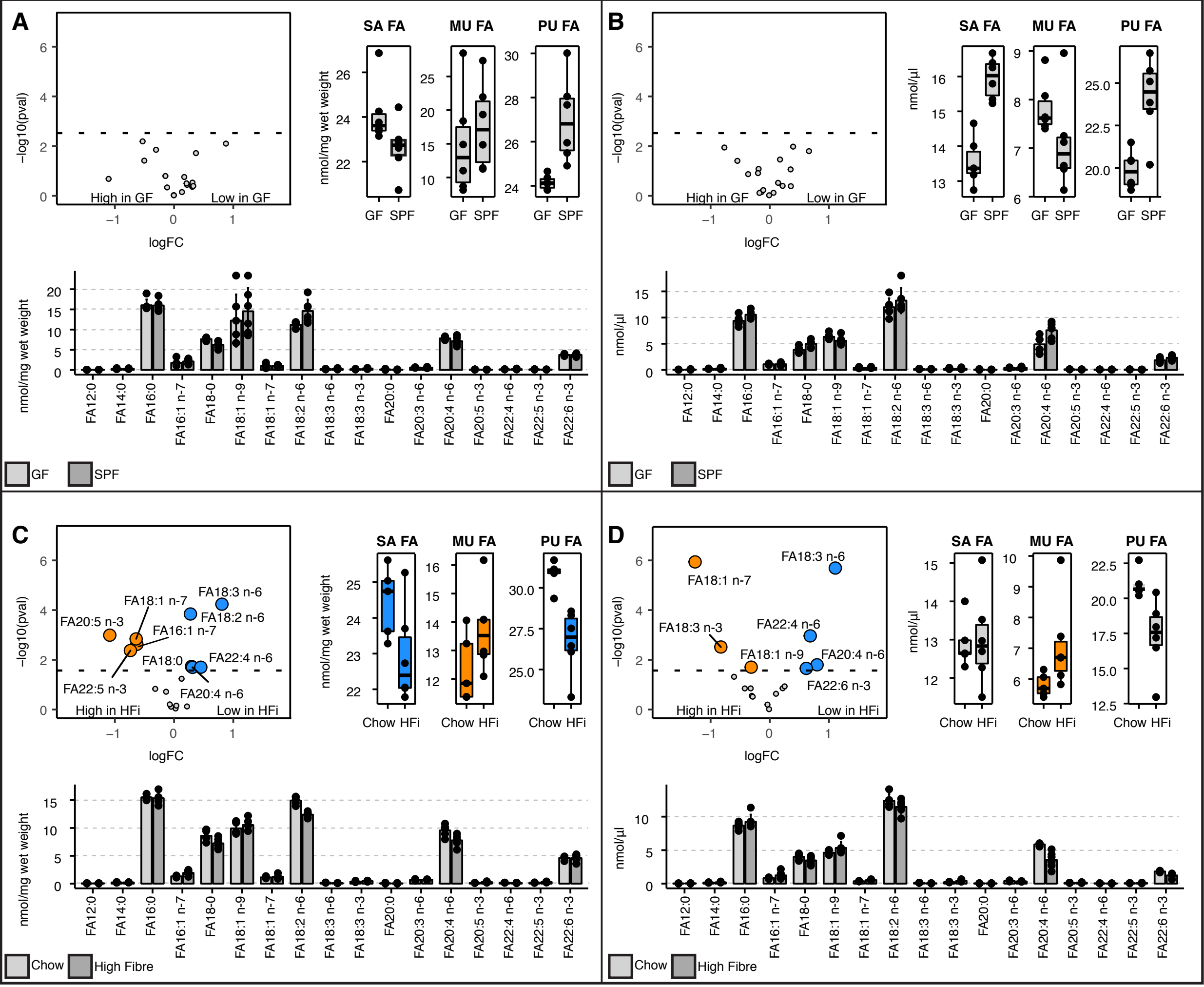

Supplementary Figure 3: Quantitative FA analyses of liver and plasma from mice fed specific diets. GF and SPF mice (n=6/group) were fed an experimental control diet containing purified cellulose (5%) as fiber source for 2 weeks. (A) Total FA in liver and (B) plasma. SPF mice (n=6/group) were fed either a chow diet (5% crude fiber) or a regular diet comparable to chow but with elevated fiber content (14% crude fiber) for 2 weeks. (C) Total FA in liver and (D) plasma. Significance and log2 fold change in individual FA species shown in volcano plot; boxplots show concentrations of saturated (SA), monounsaturated (MU), and polyunsaturated (PU) FA species; individual FA levels of the different groups as barplots. In boxplots the thick lines represent the medians, the upper and lower lines of the boxes show the 25% and 75% quartiles and the whiskers are 1.5 times the interquartile range of the data. Ch, chow; FDR, false discovery rate; GF, germfree; HFi, High Fiber; SPF, specific pathogen-free. *p<0.05, **p<0.01, ***p<0.001.
