# Supplementary material for: The gut microbiota promotes hepatic fatty acid desaturation and elongation in mice
Source: Nat Commun. 2018 Sep 14;9:3760. doi: 10.1038/s41467-018-05767-4 (PMC6138742; doi:10.1038/s41467-018-05767-4)
Supplement: Supplementary file 3 — Description of Additional Supplementary Files [file 41467_2018_5767_MOESM3_ESM.pdf]

Description of Additional Supplementary Files

File name: Supplementary Data 1:
Description: Transcriptomic data of liver samples from SPF and GF mice (n=6/6).

File name: Supplementary Data 2:
Description: Proteomic data of liver samples from SPF and GF mice (n=5/5).

File name: Supplementary Data 3:
Description: Phosphoproteomic data of liver samples from SPF and GF mice (n=5/5).

File name: Supplementary Data 4:
Description: Lipidomic data of liver samples from SPF and GF mice from experiment 1 (GF: n = 6, SPF: n = 6) and experiment 2 (SPF: n = 14; GF: n = 12)
